# Diversity of bacterial communities in the plasmodia of myxomycetes

**DOI:** 10.1186/s12866-022-02725-5

**Published:** 2022-12-22

**Authors:** Shu Li, Bao Qi, Wan Wang, Xueyan Peng, Andrey A Gontcharov, Bao Liu, Qi Wang, Yu Li

**Affiliations:** 1 Engineering Research Center of Chinese Ministry of Education for Edible and Medicinal Fungi, Jilin Agricultural University, Changchun, 130118 China; 2 Key Laboratory of Molecular Epigenetics of the Ministry of Education, Northeast Normal University, Changchun, 130024 China; 3 Federal Scientific Center of the East Asia Terrestrial Biodiversity, Far Eastern Branch of the Russian Academy of Sciences, Vladivostok, 690022 Russia

**Keywords:** Slime mold, 16S rRNA, Bacteria, Amplicon sequencing

## Abstract

**Background:**

Myxomycetes are a group of eukaryotes belonging to Amoebozoa, which are characterized by a distinctive life cycle, including the plasmodium stage and fruit body stage. Plasmodia are all found to be associated with bacteria. However, the information about bacteria diversity and composition in different plasmodia was limited. Therefore, this study aimed to investigate the bacterial diversity of plasmodia from different myxomycetes species and reveal the potential function of plasmodia-associated bacterial communities.

**Results:**

The bacterial communities associated with the plasmodia of six myxomycetes (*Didymium iridis, Didymium squamulosum, Diderma hemisphaericum, Lepidoderma tigrinum, Fuligo leviderma,* and *Physarum melleum*) were identified by 16S rRNA amplicon sequencing. The six plasmodia harbored 38 to 52 bacterial operational taxonomic units (OTUs) that belonged to 7 phyla, 16 classes, 23 orders, 40 families, and 53 genera. The dominant phyla were Bacteroidetes, Firmicutes, and Proteobacteria. Most OTUs were shared among the six myxomycetes, while unique bacteria in each species only accounted for a tiny proportion of the total OTUs.

**Conclusions:**

Although each of the six myxomycetes plasmodia had different bacterial community compositions, a high similarity was observed in the plasmodia-associated bacterial communities' functional composition. The high enrichment for gram-negative (> 90%) and aerobic (> 99%) bacteria in plasmodia suggest that myxomycetes may positively recruit certain kinds of bacteria from the surrounding environment.

**Supplementary Information:**

The online version contains supplementary material available at 10.1186/s12866-022-02725-5.

## Background

Myxomycetes are a group of eukaryotes belonging to Amoebozoa, characterized by a distinctive life cycle, including the plasmodium and fruit body stages. Most myxomycetes species are thought to have extensive distribution ranges [1]. They can feed on bacteria, algae, fungal spores, hyphae, and other organic materials [2]. Myxomycetes were estimated to account for up to 50% of total soil amoebae in soil [3], consuming a significant portion of soil bacteria [4, 5].

However, the relationship between myxomycetes and bacteria may be more complex than mere predation [6]. The study of myxomycetes-associated bacteria has long attracted the attention of researchers for revealing ecological relationships between myxomycetes and bacteria, studying the contribution of myxomycetes to the formation of the environment's microbial communities [7], and understanding better of myxomycetes nutrition requirements. In myxomycetes' life cycle, swarm cells [8–10], myxamoeba [11–13], and plasmodia [14–18] are all associated with one or several certain species of bacteria based on the culture-based method [6–8]. In view of statements that several species of bacteria were found inconstant association with different plasmodial species of myxomycetes and Acrasieae, a qualitative analysis of the microbial flora of gross cultures of plasmodia was undertaken to determine the significance of their association. The culture-based method showed that five to eight bacteria are associated with one plasmodium [19]. Two-membered culture (feeding of one living organism upon another organism which in turn thrives upon the supplied substrate) was established by Pinoy to investigate the relationship between myxomycete and bacteria [14]. According to the two-membered cultures, bacteria were necessary for myxomycete growth [15, 19, 20]. In plasmodia of *Physarum nicaraguense* Macbr. [6], only living bacteria other than heat-killed bacteria are suitable for plasmodia growth, indicating that the living bacteria probably meet myxomycetes' nutritional requirements other than just as prey. Moreover, several bacterial species, mainly in Enterobacteriaceae, can live with myxomycetes plasmodia symbiotically [21]. These results all suggest the complex relationship between myxomycetes and associated bacteria.

However, the culture-based methods may underestimate those non-cultivable bacteria, as shown in a study utilizing amplicon sequencing of 16S rRNA genes which found that 31 bacterial genera are inhabiting the fruiting bodies of the *Lycogala epidendrum *[7]. High-throughput sequencing provides an opportunity to better reveal the myxomycetes-associated bacteria diversity and may shed light on understanding the relationship between myxomycetes and bacteria. Through 16S rRNA amplicon sequencing, the bacterial community associated with plasmodia of six myxomycetes was analyzed in this study.

## Results

### Bacterial communities associated with plasmodia

**Fig. 1:**
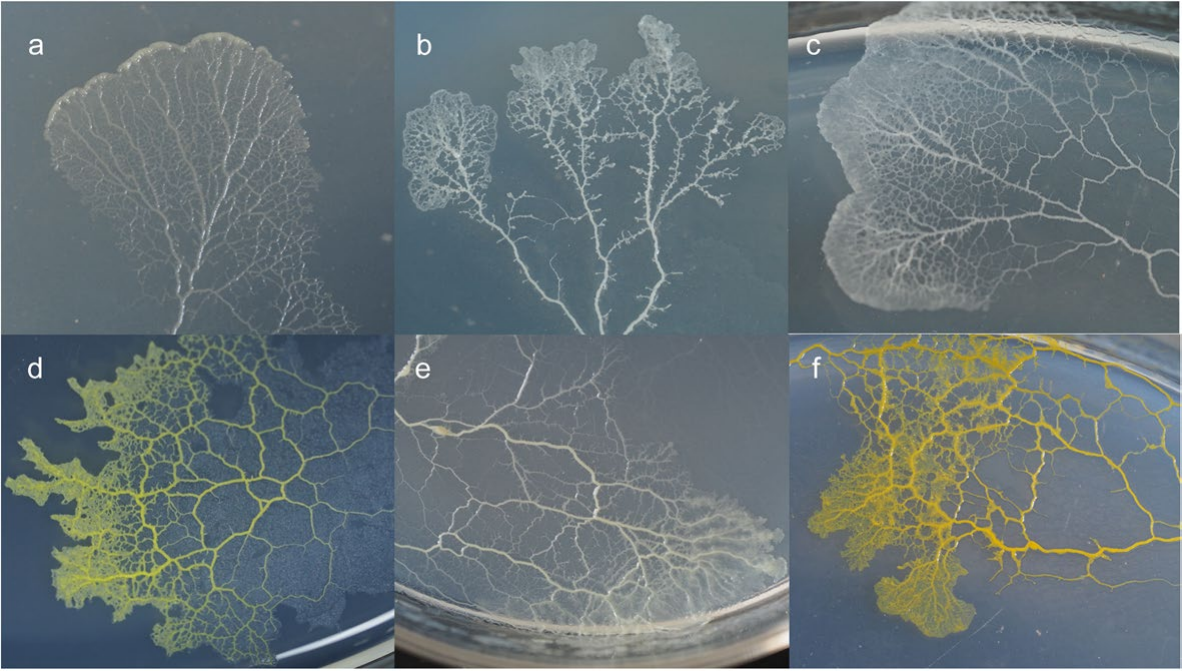
Six plasmodia cultured on oat—agar media. **a** *Didymium iridis* (Ditmar) Fr.; **b** *Didymium squamulosum* (Alb. & Schwein.) Fr.; **c** *Diderma hemisphaericum* (Bull.) Hornem.; **d** *Lepidoderma tigrinum* (Schrad.) Rostaf.; **e** *Fuligo leviderma* H. Neubert, Nowotny & K. Baumann; **f** *Physarum melleum* (Berk. & Broome) Massee

Plasmodia-associated bacterial communities of six myxomycetes (Fig. 1) were investigated by 16S amplicon sequencing. In total, 390,819 high-quality clean reads were clustered into 88 OTUs (Operational Taxonomic Units) with > 97% sequence similarity, and the plasmodia of six myxomycetes species harbored 38 to 52 bacterial OTUs. The Good's coverage score ranged from 0.9998 to 0.9999 (Table 1), suggesting that sequencing depths were adequate to describe the bacterial community.

**Table 1:** Statistic of OTUs and the Good's Coverage index in each sample

Families	Species	OTUs	The Good’s Coverage
Didymiaceae	*Didymium iridis*	50	0.9999
	*Didymium squamulosum*	43	0.9999
	*Diderma hemisphaericum*	41	0.9998
	*Lepidoderma tigrinum*	52	0.9999
Physaraceae	*Fuligo leviderma*	38	0.9999
	*Physarum melleum*	50	0.9998

**Fig. 2:**
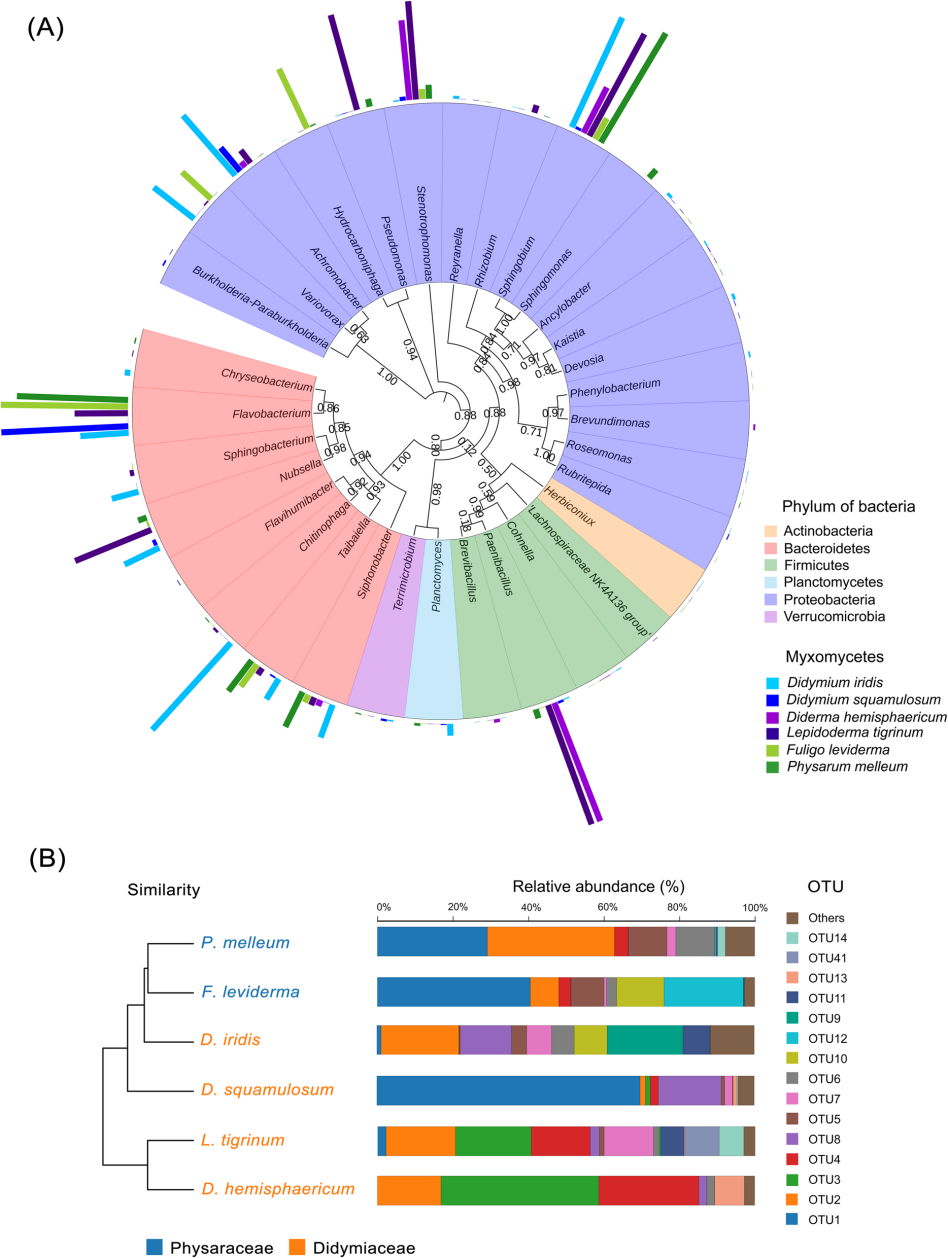
Bacterial communities associated with plasmodia. **a** Phylogenetic analysis of bacterial communities associated with six myxomycetes at the genus level. A neighbor-joining tree exhibit the bacterial genera (relative abundance above 0.1%) detected in this study. Colored bars represent the relative abundance of each genus in the six plasmodia. **b** Hierarchical clustering analysis (Weighted Unifrac UPGMA) of bacterial communities associated with each plasmodium and relative abundance of bacteria at the OTU level

The 88 bacterial OTUs belonged to 7 phyla, 16 classes, 23 orders, 40 families, and 53 genera (Table S1), and the dominant phyla were Bacteroidetes (accounted for 20.45% of the total OTUs), Firmicutes (accounted for 23.86% of the total OTUs), and Proteobacteria (accounted for 47.72% of the total OTUs) (Fig. 2a). In most cases, there were two dominant phyla (5 out of 6 myxomycetes). However, in *L. tigrinum,* there were three dominant phyla. Proteobacteria and Bacteroidetes were dominant in most myxomycetes (5 out of 6), while Flavobacteria was dominant in *L. tigrinum*. In *D. squamulosum*, *Flavobacterium* sp. (OTU1) was dominant and accounted for 66.84% of all OTUs, while in *P. melleum* and *F. leviderma*, there were two dominant OTUs (*Flavobacterium* sp., OTU1 and *Sphingobium* sp., OTU2) and accounting for 58.29%-59.95% of all OTUs. In the other three plasmodia, there were more than three dominant OTUs, and the most abundant OTU (*Sphingobium* sp., OTU2) in *D. iridis* only accounted for 18.51% of all OTUs. Bacteria communities in *P. melleum* and *F. leviderma* resembled each other and were clustered together. Bacteria communities in *L. tigrinum* and *D. hemisphaericum* formed another separate cluster. *D. iridis,* and *D. squamulosum* were more closely related to *L. tigrinum* and *D. hemisphaericum*. However, the bacterial communities in *D. iridis* and *D. squamulosum* showed a closer relationship to *P. melleum* and *F. leviderma* (Fig. 2b).

**Fig. 3:**
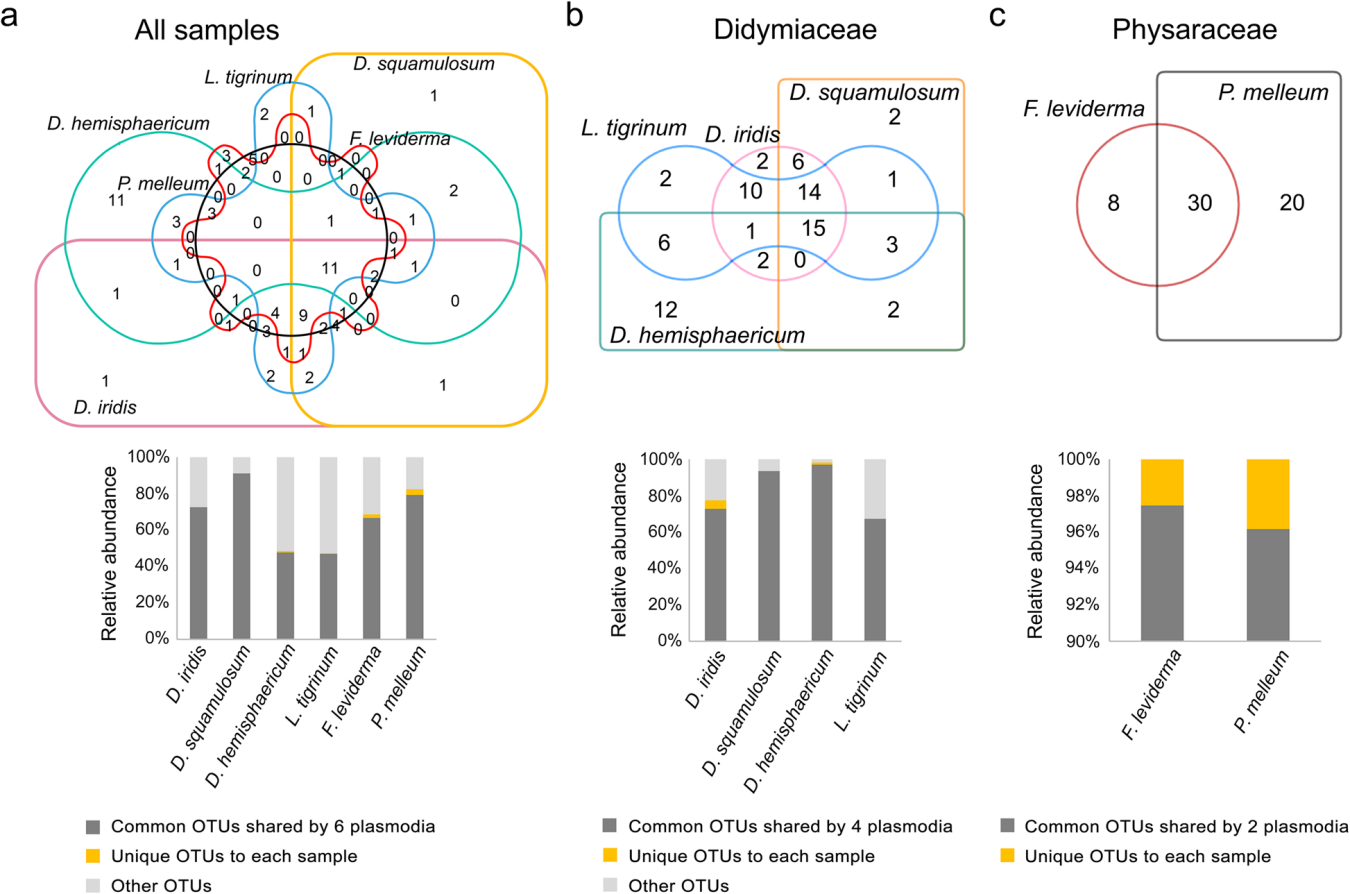
Venn diagrams of overlapping OTUs. Colored bars represented the relative abundance of common and unique OTUs

Although the six myxomycetes plasmodia had different bacterial community compositions, most OTUs were shared by the six myxomycetes, especially for those abundant OTUs (Fig. 3a). Only a minor proportion of OTUs (0.02% to 3.31%) was unique to each species. Specifically, 70% (35 out of 50 OTUs) of the *D. iridis* associated bacterial OTUs and 81.40% (35 out of 43 OTUs) of the *D. squamulosum* associated bacteria OTUs were shared by the two species which belonged to the same genus. Three species of Didymium and Diderma shared 15 OTUs, 29 OTUs were shared by three species of *Didymium* and *Lepidoderma*, and Diderma and Lepidoderma shared 25 OTUs. Among the shared OTUs, most of the OTUs (> 80%) with an abundance of above 1% (> 80%) were included (Fig. 3b). In Physaraceae, 78.95% (30 out of 38 OTUs) of *F. leviderma* associated bacterial OTUs and 60% (30 out of 50 OTUs) of *P. melleum* associated bacterial OTUs were shared by the two species (Fig. 3c). Most of the associated bacterial OTUs in the two families were also overlapped.

### Core microbiota shared by all the myxomycetes

There were 11 OTUs shared by all six species, most of which had a relatively high abundance. The 11 shared OTUs of all plasmodium belonged to 11 families, 9 orders, and 7 classes (Table S1) and were defined as core microbiota. The percentages of the total community covered by the shared OTUs ranged from 93% (*D. iridis*) to 99% (*D. hemisphaericum* and *F. leviderma*).

### Microbiota phenotypes and functions prediction

**Fig. 4:**
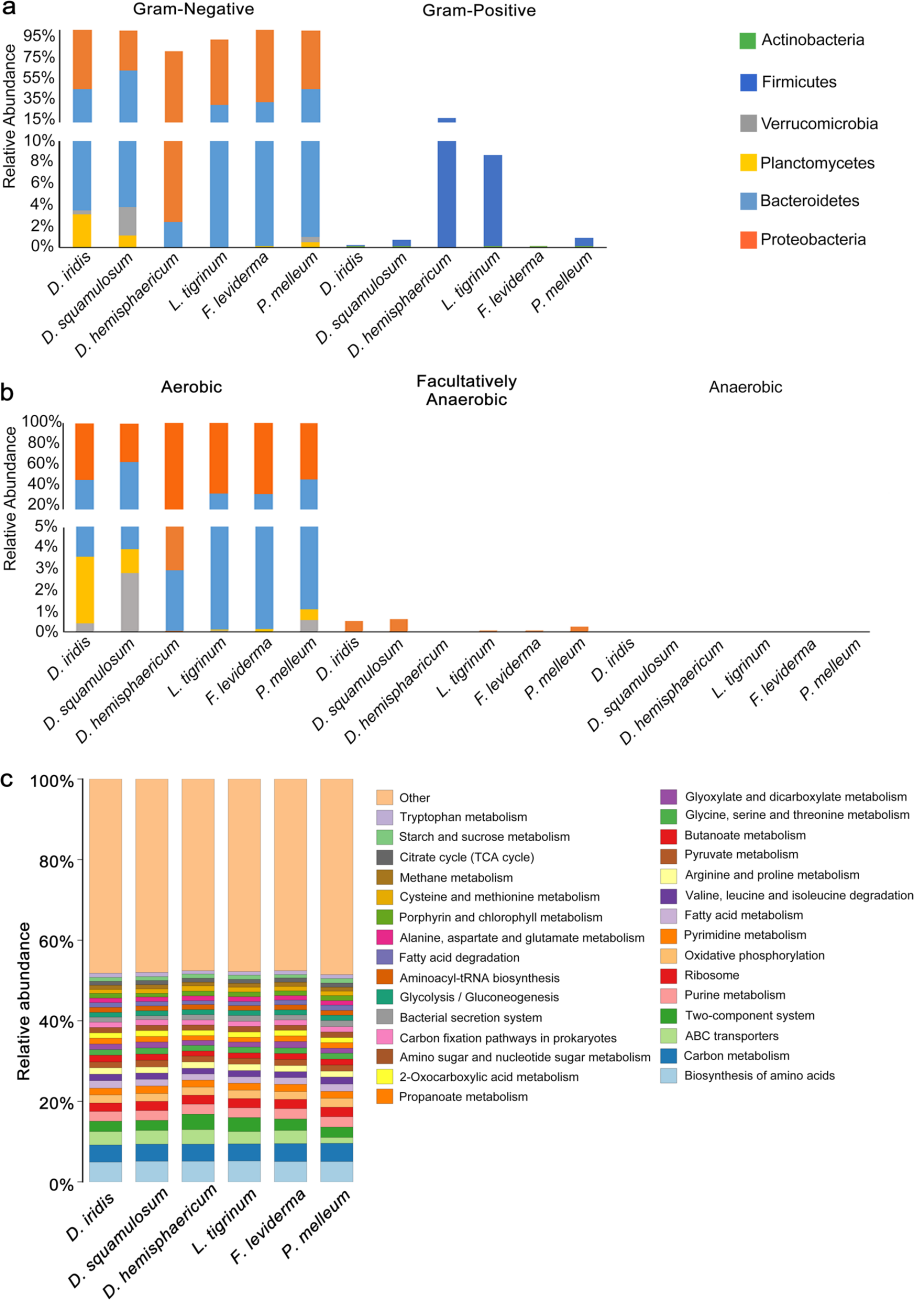
Phenotypes and function of bacterial communities predicted in 6 myxomycetes. **a** Gram bacterial classification predicted by BugBase. **b** Oxygen utilization predicted by BugBase. **c** Top 30 enriched KEGG function at level 3 prediction of bacterial communities associated with six myxomycetes by PICRUSt2

According to the Bugbase prediction (Fig. 4a, Table S2), the six plasmodia were predominately associated with Gram-negative bacteria, accounting for more than 90% of the total bacterial community. Moreover, nearly all (> 99%) of the plasmodia-associated bacteria were aerobic (Fig. 4b, Table S3). PICRUSt2 was implemented to gain insight into bacterial community function. KEGG pathways were demonstrated in 3 levels; the top 30 most abundant KEGG pathways were represented, and the associated bacteria of six myxomycetes species exhibited a remarkable similarity in the functional composition (Fig. 4c, Table S4). Functions in level 1 include metabolism (72.6%-76.2%), genetic information processing (8.4%-9.3%), environmental information processing (6.3%-8.7%), human diseases (3.3%-4.2%), cellular processes (2.4%-3.5%), and organismal systems (1.1%-1.4%). Metabolism is one of the most important functions (Fig. 4c, Table S4). The most abundant KEGG pathways related to metabolism are carbohydrate metabolism and amino acid metabolism in level 2, and in level 3, the top 2 most abundant KEGG pathways are the biosynthesis of amino acids and carbon metabolism (Fig. 4c, Table S4).

## Discussion

It has long been believed that one or two bacterial species were closely associated with one plasmodium [6, 22]; by using six myxomycetes plasmodia belonging to five genera and two families, our results illustrated that 38 to 52 bacterial OTUs were associated with one myxomycetes species, and in most cases, there were more than one dominant OTUs. Our results showed that plasmodia harbored much more abundant bacteria than previously thought; a similar result has been reported in the fruit body of *Lycogala epidendrum* [7]. These plasmodia-associated bacteria were predominately gram-negative (> 90%) and aerobic (> 99%) (Fig. 4a, b), which is in accordance with previous research that the plasmodia-associated bacteria are almost gram-negative [19]. The high enrichment for gram-negative (> 90%) and aerobic (> 99%) bacteria in plasmodia suggest that myxomycetes may positively recruit certain kinds of bacteria from the surrounding environment. Furthermore, although they belong to 7 phyla, the associated bacteria of six myxomycetes species exhibited a remarkable similarity in the functional attributes (Fig. 4c, Table S4). These findings imply that the relationship between myxomycetes and their associated bacteria is far more complicated and may provide similar functional capacity for myxomycetes other than just as a food source [23].

Enterobacteriaceae is reported to associate with myxomycetes; however, we did not identify any Enterobacteriaceae in this study [21], which might be attributed to the different myxomycetes species used by different research. Although the relative abundances of the bacteria in each myxomycetes species were substantially different, the six myxomycetes plasmodia shared similar bacterial groups, which is also in good accordance with previous research results [19]. The shared OTUs of all plasmodia accounted for more than 93% of the total OTUs, while the unique bacteria in each myxomycetes species only accounted for a tiny proportion (0.02% to 3.31%) of the total OTUs. This result differed from those in coral, where the abundance of main symbiotic microbial communities was extremely low [24].

## Conclusion

Bacterial communities of 6 plasmodia of Physarales were investigated, and the results showed that plasmodia harbored much more abundant bacteria than previously thought. Most OTUs were shared among the six myxomycetes, while unique bacteria in each species only accounted for a minor proportion of the total bacteria. Furthermore, these plasmodia-associated bacteria were predominately Gram-negative and aerobic, which suggests that myxomycetes may positively recruit certain kinds of bacteria from the surrounding environment. Finally, although these plasmodia-associated bacteria belong to 7 phyla, they exhibited a remarkable similarity in their functional attributes, which implies that the relationship between myxomycetes and their associated bacteria is far more complicated and may provide similar functional capacity for myxomycetes other than just as a food source.

## Materials and methods

### Artificial culture of plasmodia

Fruiting bodies of *Didymium iridis, Didymium squamulosum, Diderma hemisphaericum, Lepidoderma tigrinum, Fuligo leviderma,* and *Physarum melleum* were collected from the field (Table 1). The morphological features of all the specimens were observed under a light microscope (LM, Zeiss Axio Scope A1, Germany). For LM observation, spores, capillitia, and lime nodes of the specimen were mounted in a drop of Hoyer's medium on glass slides [25]. The 75% (v/v) ethanol was used to clean the fruiting bodies' surface with complete peridium, and the spores were collected using sterile tweezers after peeling off the peridium. The collected spores were mixed with sterile water in a 2-mL plastic tube. A few drops of the spore suspension were scattered on the surface of oat agar media in 9-cm Petri-dishes, followed by incubation in the dark at 25 ℃. After spore germination, the six myxomycetes' plasmodia were cultured on the oat agar media in 9-cm Petri dishes, then incubated in the dark at 25 °C [26]. Several plasmodia germinated from spores of one specimen were cultivated for each species. The plasmodia were regularly sub-cultured on sterile oat agar media. When the plasmodia had grown and occupied most of the medium surface, they were transferred to a fresh sterile oat agar media. The fresh sub-culturing was performed every 5 to 7 days for three months (Fig. 1).

### DNA extraction and amplicon sequencing

According to the manufacturer's protocols, under aseptic conditions, genomic DNA was extracted from plasmodia using the Universal Genomic DNA Kit (CWBIO, China) according to the manufacturer's protocols. About 200 mg of plasmodia (collected from several individual plasmodia) were used for DNA extraction. DNA purity was examined on 1% agarose gels. NanoPhotometer spectrophotometer was used to detect sample purity; Qubit2.0 Flurometer was used to measure DNA sample concentration. The V3-V4 hypervariable region of the bacterial 16S ribosomal RNA gene was amplified by PCR (94 °C for 2 min, followed by 30 cycles at 94 °C for 30 s, 55 °C for 30 s, and 72 °C for 30 s, and a final extension at 72 °C for 10 min) using primers 341F 5'–CCTACGGGNGGCWGCAG–3' and 805R 5'–GACTACHVGGGTATCTAATCC–3' [27], where the barcode was an eight-base sequence unique to each sample. PCR was performed in triplicate with 50 μL mixture containing 5 μL of 10 × Ex Taq Buffer (Mg^2+^ plus), 4 μL of 2.5 mM dNTPs, 1 μL of each primer (20 μM), 0.25 μL of TaKaRa Ex Taq (5 U/μL), and 2.5 ng of the template DNA. Amplicon sequencing targeting 16S V3–V4 region was performed by Annoroad company based on an Illumina HiSeq platform. Roughly 50,000, 250 bp paired-end reads were generated for each sample. Original data have been deposited into the NCBI SRA database with the accession number PRJNA600342.

### Data analyses

Paired-end reads were assigned to each sample based on their unique barcode and truncated by cutting off the bar code and primer sequence. Then, the reads were merged using FLASH v 1.2.11 (http://ccb.jhu.edu/software/FLASH/) [28]. Quality filtering was performed with default parameters using Trimmomatic v 0.33 [29] and UCHIME v8.1 [30], according to the quality-controlled process of QIIME(v1.7.0, http://qiime.org/index.html) [31].

Sequence analyses were performed using Uparse v7.0.1001 software (http://drive5.com/uparse/) [32]. Sequences with ≥ 97% similarity were assigned to the same OTUs, and a representative sequence for each OTU was selected for further annotation. For each representative sequence, the SILVA database (http://www.arb-silva.de) [33] was used to annotate taxonomic information with RDP Classifier v2.2 (http://sourceforge.net/projects/rdpclassifier/) [34].

The abundance information of OTUs was normalized by a standard of the sequence number corresponding to the sample with the least sequences using BMKCloud (www.biocloud.net). Subsequent analyses of alpha diversity were performed based on the normalized data, and the Good's coverage index was used to check for adequate sampling depth.

For predicting microbial phenotypes and functions, metagenomes were inferred from the 16S rRNA data using the BugBase tool (https://bugbase.cs.umn.edu/) [35] and PICRUSt2 [36] after reclassifying OTUs with the Greengenes database (http://greengenes.lbl.gov) [37]. In order to study the phylogenetic relationship among different OTUs, and the difference in the dominant species in different samples (groups), a phylogenetic tree was constructed using iTOL (Interactive Tree Of Life) [38].

## Supplementary Information

**Additional file 1:** **Supplementary Table S1.** Taxonomy annotation of OTUs and their abundance in each species.

**Additional file 2:** **Supplementary Table S2.** Composition of Gram-negative and positive bacteria in 6 plasmodia. **Supplementary Table S3.** Composition of aerobic, facultatively anaerobic and anaerobic bacteria in 6 plasmodia.

**Additional file 3: Supplementary Table S4.** The top 30 most abundant KEGG pathways and top 9 KO functions of bacterial communities associated with six myxomycetes by PICRUSt2.

## Data Availability

Original data have been deposited into the NCBI SRA database with the accession number PRJNA600342.
