# Supplementary material for: Diversity of bacterial communities in the plasmodia of myxomycetes
Source: BMC Microbiol. 2022 Dec 22;22:314. doi: 10.1186/s12866-022-02725-5 (PMC9773492; doi:10.1186/s12866-022-02725-5)
Supplement: Supplementary file 2 — Additional file 2: Supplementary Table S2. Composition of Gram-negative and positive bacteria in 6 plasmodia. Supplementary Table S3. Composition of aerobic, facultatively anaerobic and anaerobic bacteria in 6 plasmodia. [file 12866_2022_2725_MOESM2_ESM.docx]

**Supplementary Table S2.** Composition of Gram-negative and positive bacteria in 6 plasmodia

| Phylum of bacteria | | *D. iridis* | *D. squamulosum* | *D. hemisphaericum* | *L. tigrinum* | *F. leviderma* | *P. melleum* |
| --- | --- | --- | --- | --- | --- | --- | --- |
| Gram-negative bacteria | Bacteroidetes | 42.49% | 59.33% | 2.36% | 31.35% | 34.06% | 45.35% |
|  | Proteobacteria | 53.86% | 36.03% | 78.12% | 59.85% | 65.68% | 52.70% |
|  | Verrucomicrobia | 0.36% | 2.75% | 0 | 0.05% | 0.01% | 0.52% |
|  | Planctomycetes | 3.11% | 1.15% | 0 | 0.04% | 0.11% | 0.52% |
| Gram-positive bacteria | Firmicutes | 0.08% | 0.60% | 19.52% | 8.61% | 0.05% | 0.80% |
|  | Actinobacteria | 0.10% | 0.14% | 0 | 0.10% | 0.10% | 0.11% |

**Supplementary Table S3.** Composition of aerobic, facultatively anaerobic and anaerobic bacteria in 6 plasmodia

| Phylum of bacteria | | *D. iridis* | *D. squamulosum* | *D. hemisphaericum* | *L. tigrinum* | *F. leviderma* | *P. melleum* |
| --- | --- | --- | --- | --- | --- | --- | --- |
| Aerobic | Proteobacteria | 52.20% | 35.50% | 97.07% | 65.30% | 65.71% | 52.08% |
|  | Bacteroidetes | 43.73% | 59.99% | 2.93% | 34.54% | 34.16% | 46.63% |
|  | Verrucomicrobia | 0.37% | 2.78% | 0 | 0.05% | 0.01% | 0.53% |
|  | Planctomycetes | 3.20% | 1.16% | 0 | 0.05% | 0.11% | 0.53% |
| Facultatively anaerobic | Proteobacteria | 0.49% | 0.58% | 0 | 0.06% | 0.02% | 0.22% |
| Anaerobic | - | 0 | 0 | 0 | 0 | 0 | 0 |
